# Evidence mapping of traditional Chinese medicine in diabetic peripheral neuropathy treatment

**DOI:** 10.3389/fphar.2024.1325607

**Published:** 2024-03-28

**Authors:** Yujie Fu, Yiming Wang, Zhenghong Li, Ke Huang, Yating Gao, Shanqiong Xu, Qingna Li, Xingfang Liu, Guangde Zhang

**Affiliations:** ^1^ Graduate School, Beijing University of Chinese Medicine, Beijing, China; ^2^ Graduate School, China Academy of Chinese Medical Sciences, Beijing, China; ^3^ Research Department, Swiss University of Traditional Chinese Medicine, Bad Zurzach, Switzerland; ^4^ Institute of Endocrinology, Xiyuan Hospital, China Academy of Chinese Medical Sciences, Beijing, China; ^5^ Institute of Clinical Pharmacology, Xiyuan Hospital, China Academy of Chinese Medical Sciences, Beijing, China

**Keywords:** diabetic peripheral neuropathy, diabetes, complications, traditional Chinese medicine, randomized controlled trial, systematic review, evidence mapping

## Abstract

**Objective:** Diabetic peripheral neuropathy (DPN) stands as a crucial complication of diabetes, significantly affecting patients’ quality of life. This study aims to elucidate the evidence distribution from clinical randomized controlled trials (RCTs) on DPN treatment with traditional Chinese medicine (TCM) through evidence mapping.

**Methods:** A comprehensive search was conducted from January 2017 to October 2022 in databases such as Wanfang (China Online Journals), CNKI (China National Knowledge Infrastructure), VIP (China Science and Technology Journal Database), SinoMed (Chinese Biomedical Literature Database), PubMed, Web of Science, and Cochrane Library. Literature related to the treatment of DPN with TCM was selected. From the 1,229 RCTs identified over the past 6 years, relevant data were extracted. The evidence mapping approach was utilized, and trends in publications, study scales, intervention types, and evaluation indicators were analyzed using descriptive text combined with tables and bubble charts.

**Results:** Research on the treatment of DPN with TCM is extensive. The publication trend remains relatively stable with predominantly smaller sample sizes. The main treatments encompass oral Chinese medicine and traditional external treatments. The most common evaluation indicators are neurophysiological, efficiency rate, symptom signs, neuropathy scores, and traditional Chinese symptoms, with less focus on psychological status and the ankle-brachial index (ABI).

**Conclusion:** Shedding light on contemporary research, this study explores the current RCTs evaluating TCM’s efficacy in treating DPN. The findings not only highlight the potential role of TCM in addressing diabetic complications but also underscore areas that could benefit from refined research approaches, expanded intervention methods, and broader assessment criteria. Our observations aim to inform and inspire future research directions and clinical practices concerning TCM’s role in managing diabetes-associated complications.

## 1 Introduction

Diabetic Peripheral Neuropathy (DPN) is a prevalent neurological complication of diabetes. Between 11% and 49% of prediabetic patients can exhibit symptoms, even in the disease’s preliminary stages (Ziegler et al., 2008; Lee et al., 2015; Won et al., 2017). After a decade of affliction, 60%–90% of diabetic individuals might display various degrees of neuropathic impairments, nearly half of which are categorized as DPN (Tesfaye et al., 2010). These complications encompass challenges such as balance difficulties, excruciating neuropathic pain, and, in advanced instances, irreversible nerve injuries leading to diabetic foot ulcers and possible amputations (Morrison et al., 2012; Selvarajah et al., 2019). On an individual level, age remains a persistent risk factor for DPN (Liu et al., 2019). As patients age and disease duration elongates, the likelihood of developing DPN intensifies (Cabezas-Cerrato, 1998). On a macro scale, with rapid economic growth coupled with accelerated aging populations, the global number of elderly diabetic patients and those with DPN is anticipated to continually ascend (Yang et al., 2010; Powell et al., 2012; Xu et al., 2013; Wang et al., 2023). In 2019, the population of elderly diabetic individuals (aged 65–99) was approximately 135.6 million, accounting for 19.3%. This number is projected to soar to 195.2 million by 2030 (Brussels, 2019; Saeedi et al., 2019). With many elderly already having underlying conditions, the onset of DPN may precipitate more grave consequences and comorbidities. Consequently, efficacious and economical prevention and treatment strategies, alongside evaluation metrics, are paramount for this group. Traditional Chinese Medicine (TCM) has demonstrated certain therapeutic efficacy in treating DPN, offering diverse treatments that alleviate symptoms and delay disease progression through multiple targets and pathways (Yang et al., 2022).

With the burgeoning emphasis on high-quality clinical trial research within the TCM realm, there has been an influx of randomized controlled trials (RCTs) exploring diverse TCM interventions for DPN. Yet, an overarching synthesis of evidence in this area is notably absent. Evidence mapping, a methodological tool that traces its origins to Yale University’s research on complementary and alternative medicine, mandates a comprehensive retrieval and systemic summation of research attributes and outcomes. By utilizing graphical elucidations, it furnishes a lucid and precise portrayal of the research field’s evidence, advancements, and existing challenges (Katz et al., 2003; Li et al., 2011; Schmucker et al., 2013), enhancing the efficacy and applicability of research in the area. This serves researchers, guideline creators, clinical physicians, and other stakeholders (Bragge et al., 2011; Miake-Lye et al., 2016; Li et al., 2019). Our study collates and analyzes RCTs from the past 6 years on multi-method TCM treatments for DPN. By integrating charts with evidence mapping, we offer a comprehensive view of the current state of research in the domain. This also encapsulates its limitations, contributing towards the enhancement of research quality in the field (Bastian et al., 2010), aiming to bridge the gap in research synthesis on evidence.

## 2 Methods

### 2.1 Data sources and searches

We systematically searched several databases including Wanfang, China National Knowledge Infrastructure (CNKI), VIP, SinoMed, PubMed, Web of Science, and Cochrane Library for studies on traditional Chinese medicine (TCM) interventions in diabetic peripheral neuropathy (DPN). Considering the increasing attention to TCM treatment aspects in the DPN field in 2017, this approach allowed us to collect a large number of relevant time newer publications to provide a comprehensive reference for future research. The search timeframe was from January 2017 to October 2022, including only Chinese and English articles. Both MeSH terms and free-text terms were used. The search terms included but were not limited to “糖尿病性周围神经病变”, “糖尿病周围神经病变”, “中医”, “"中药”, and their English counterparts “Diabetes peripheral neuropathy”, “DPN”, “traditional Chinese medicine”, “herbal medicine”, and “TCM”. The search strategy for Web of Science was: [TS = (Diabetes peripheral neuropathy) OR TS = (DPN)] AND [TS = (traditional Chinese medicine) OR TS=(herbal medicine) OR TS = (TCM)].

### 2.2 Study selection

#### 2.2.1 Inclusion criteria

The studies considered for inclusion were clinical RCTs that investigated TCM treatments for DPN. Participants in these studies could be patients diagnosed with DPN, with no restrictions regarding age, gender, disease duration, or comorbidities. The interventions examined in the selected studies could encompass a range of treatments including oral Chinese herbal decoctions, granules, proprietary Chinese medicines, Chinese medicine injections, acupuncture, foot baths, massage, fumigation washing, integrated TCM care, combined TCM therapies, etc.

#### 2.2.2 Exclusion criteria

Studies were excluded if they were duplicated publications, inaccessible full-texts, animal or cellular experiments.

### 2.3 Data extraction and quality assessment

All identified references were imported into NoteExpress3.7.0 for management. A group of five researchers conducted the literature screening, with one appointed as the team leader. To ensure consistency, 100 papers were pre-screened as a calibration exercise before the main screening, and uniform screening criteria were established. Titles and abstracts were initially screened for relevance. Potentially relevant papers were further assessed by reading the full text. Discrepancies during the screening were resolved through discussions led by the team leader. Relevant information was extracted using a standardized form, which included publication year, authors, sample size, trial period, combined drugs, interventions (categorized as oral Chinese medicine, proprietary medicine, TCM external treatments, herbal injections, integrated TCM care, multi-therapy combination, and others), and outcome measures.

### 2.4 Classification of TCM interventions

To provide a clearer picture of TCM treatments for DPN, we categorized the TCM interventions from the included studies into seven types: oral Chinese medicines (including herbal decoctions and granules), proprietary Chinese medicines, external TCM therapies (like acupuncture, foot baths, massages, and fumigation), comprehensive TCM nursing, multi-therapy combinations, herbal injections, and others (which covers TCM exercise therapy and music therapy, etc.).

### 2.5 Data synthesis and analysis

Descriptive statistics were presented both in text and graphical forms. Graphs were plotted using Origin 2021 software, with trend developments represented using line graphs, category distributions in pie charts, and evidence distributions in bubble charts (Tian et al., 2019).

## 3 Results

### 3.1 Study selection

**FIGURE 1:**
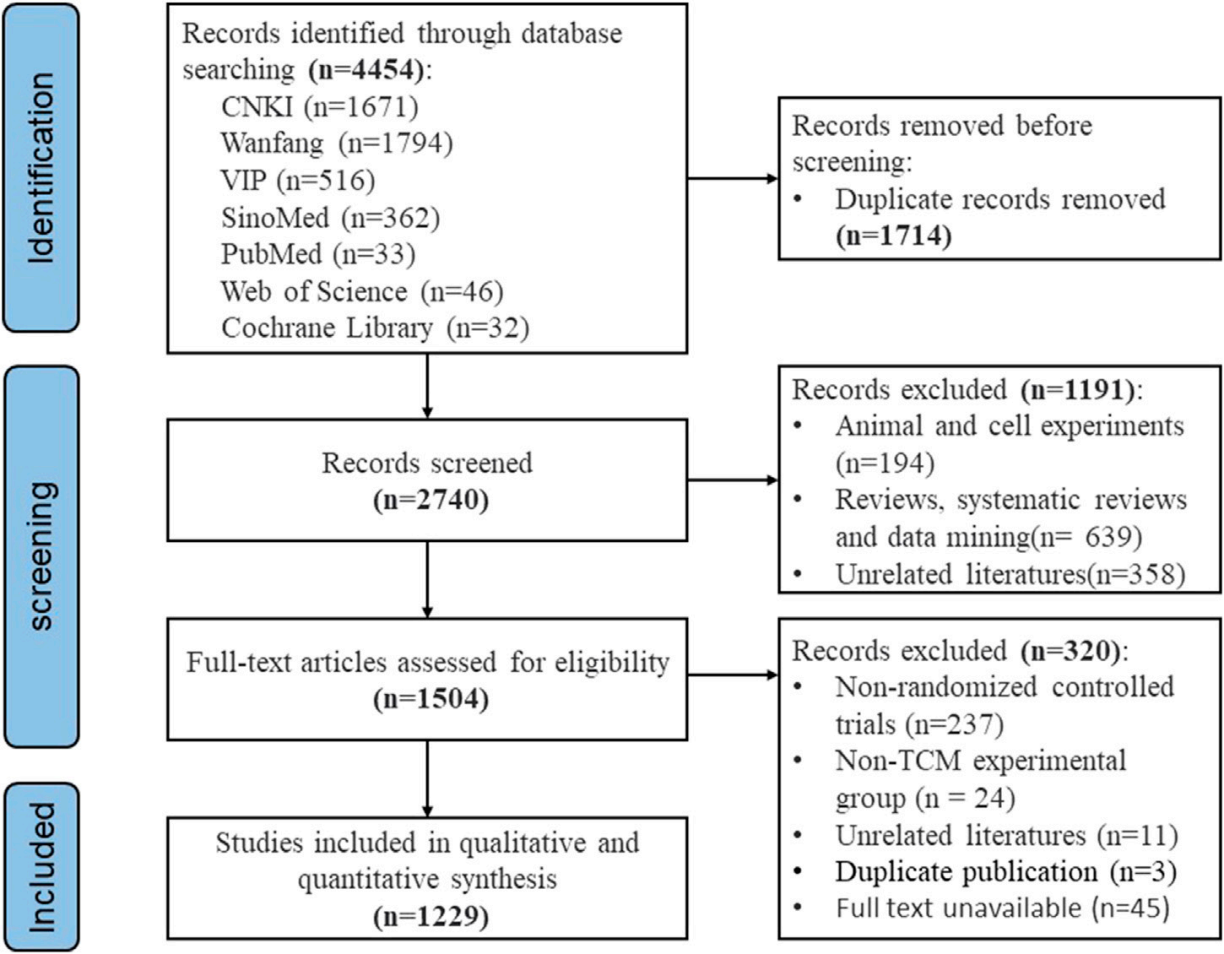
PRISMA flow diagram of literature selection process.

A total of 4,454 articles published over the past 6 years were identified through database searches. Of these, 1,229 RCTs on TCM interventions for DPN were included. The literature screening process and results are depicted in Figure 1.

### 3.2 Publication trend

**FIGURE 2:**
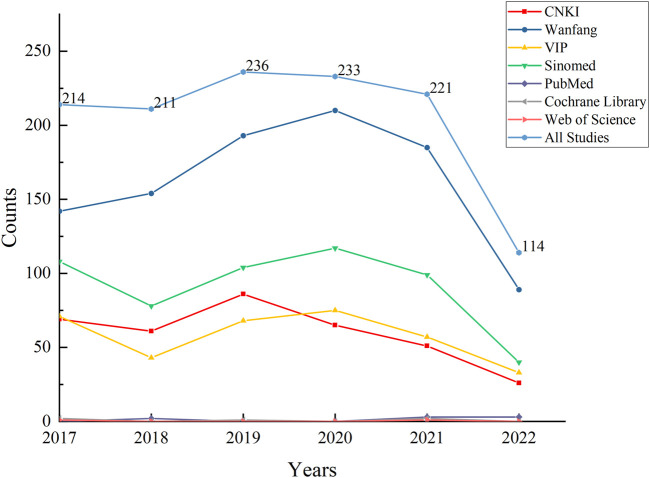
Overall publication trends of RCTs on TCM interventions for diabetic peripheral neuropathy over the past 6 years versus publication trends across databases.

From January 2017 to October 2022, we analyzed 1,229 published RCTs of TCM interventions for DPN. Over the five complete years (2017–2021) included, the annual average number of publications was 223, with no significant fluctuations, indicating a plateau in the number of publications. It is evident that Wanfang has indexed the most articles on TCM interventions for DPN. This trend is illustrated in Figure 2.

### 3.3 Analysis of sample size

An assessment of the scale of the included RCTs revealed a range in sample size from a minimum of 28 to a maximum of 900. The majority of studies had a sample size between 50 and 100, followed by studies with a sample size between 100 and 200. Detailed distributions are presented in Table 1.

**TABLE 1:** Sample sizes of RCTs on TCM interventions for diabetic peripheral neuropathy over the past six years.

Sample size	Number of articles	Proportion (%)
≤50	72	5.86
50–100	891	72.50
100–200	239	19.45
200–500	23	1.87
500–1,000	4	0.33

### 3.4 Positive drug usage

Of the 1,229 included studies, 775 (63.06%) explicitly used positive agents, including mecobalamin, thioctic acid, and prostaglandins. Another 73 studies (5.94%) employed drugs described with phrases like “neurotrophic and circulatory improvement.” In contrast, 381 studies (31.00%) either did not use or did not mention positive drugs. These agents generally showed various degrees of improvement in DPN patients’ symptoms and signs (Zhang and Ning, 2008; Han et al., 2012; Shin et al., 2013; Ma et al., 2022).

Mecobalamin, an active Vitamin B12 formulation widely used in many parts of the world for DPN treatment, promotes nucleic acid and protein synthesis within neurons. It significantly aids myelin formation and axonal regeneration, repairing damaged nerve cells and improving nerve conduction velocity, ultimately enhancing symptom signs (Sawangjit et al., 2020; Branch Group Of Neurological Complications, 2021).

Thioctic acid, a potent antioxidant, acts by inhibiting lipid peroxidation, enhancing blood flow in neurotrophic vessels, and boosting the activity of the Na + -K + -ATPase enzyme. It directly neutralizes reactive oxygen clusters and free radicals, protecting endothelial function (Ziegler et al., 2011; Han et al., 2012; Branch Group Of Neurological Complications, 2021).

Prostaglandins, physiologically active unsaturated fatty acids, and related compounds can increase cyclic adenosine monophosphate (cAMP) levels in vascular smooth muscle cells, relaxing vascular smooth muscles, reducing blood viscosity, and improving microcirculation (Branch Group Of Neurological Complications, 2021).

### 3.5 The distribution of TCM therapies

**FIGURE 3:**
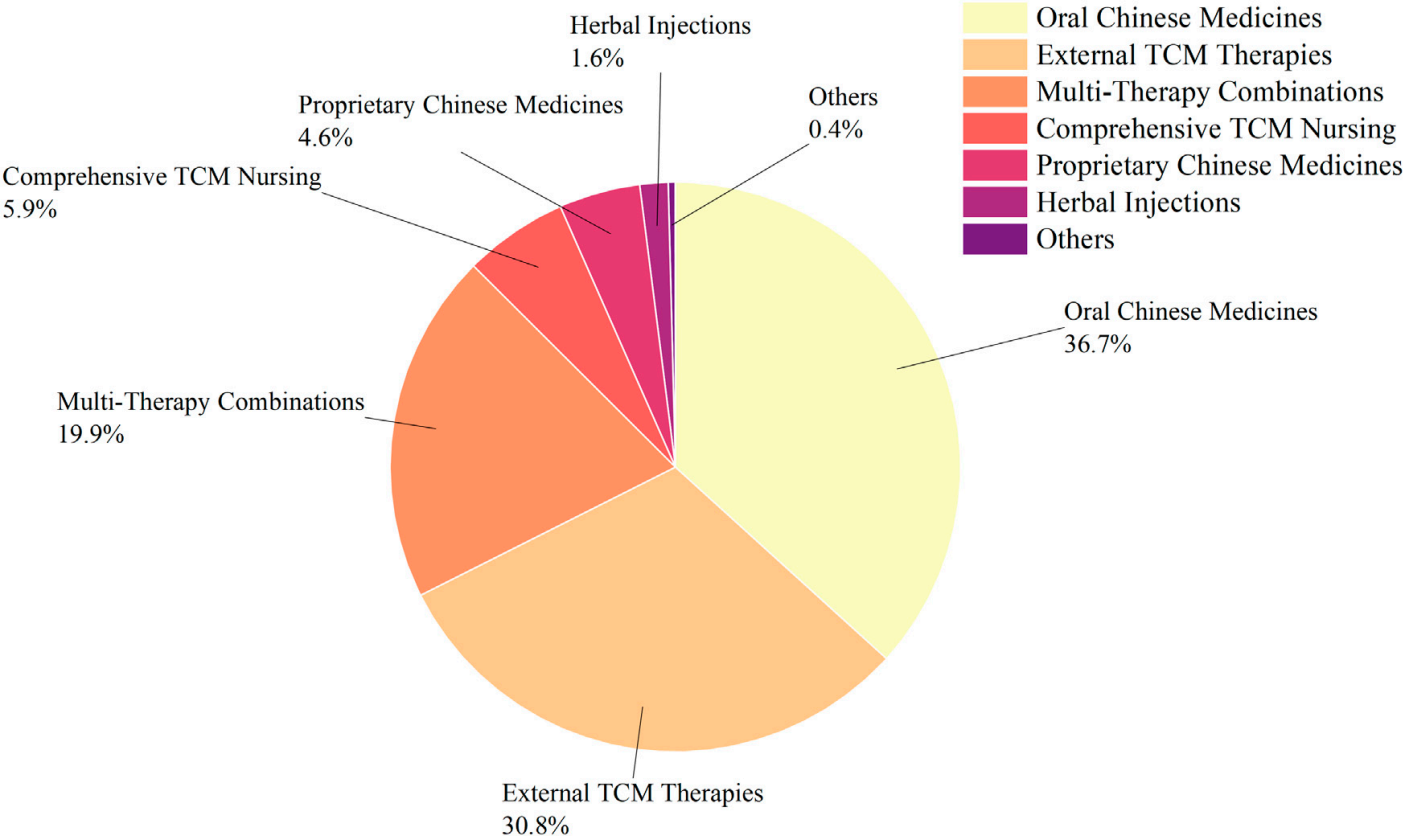
Classification of TCM interventions in RCTs for DPN treatment over the past 6 years.

Oral Chinese medicine research dominated with 453 studies (36.86%), followed by external TCM therapies with 378 (30.76%). The remaining categories included multi-therapy combinations (244 studies), comprehensive TCM nursing (73), proprietary Chinese medicines (57), herbal injections (19), and others (5). The distribution is illustrated in Figure 3.

Among the 453 studies with oral Chinese medicine as an intervention: The most frequently occurring interventions (with a frequency of ≥5) are, in descending order: Huangqi Guizhi Wuwu Decoction, Buyang Huanwu Decoction, Danggui Sini Decoction, Duhuo Jisheng Decoction, and Taohong Siwu Decoction.

For the 378 studies involving external TCM therapies: The most frequent interventions (with a frequency of ≥5) are, in descending order: acupuncture, foot bath, fumigation combined with external washing, external application, moxibustion, foot bath combined with massage, acupoint injection, external wash, external application combined with acupuncture, foot bath combined with external application, iontophoresis, foot bath combined with acupuncture, fumigation, fumigation combined with external washing and acupuncture, massage, foot bath combined with acupoint injection, and topical rubbing.

A detailed breakdown of the frequencies for each intervention is presented in Table 2. A comprehensive overview of oral Chinese medicines can be found in Tables 3, 4.

**TABLE 2:** Frequency of various intervention methods.

Types of interventions	Intervening measure	Counts
Oral Chinese Medicine	Huangqi Guizhi Wuwu Decoction	64
	Buyang Huanwu Decoction	23
	Danggui Sini Decoction	22
	Duhuo Jisheng Decoction	6
	Taohong Siwu Decoction	5
External Treatment of TCM	Acupuncture	68
	Foot Bath	59
	Fumigation Combined With External Washing	46
	External Application	28
	Moxibustion	14
	Foot Bath Combined With Massage	11
	Acupoint Injection	11
	External Wash	11
	External Application Combined With Acupuncture	11
	Foot Bath Combined With External Application	9
	Iontophoresis	8
	Foot Bath Combined With Acupuncture	8
	Fumigation	7
	Fumigation Combined With External Washing And Acupuncture	6
	Massage	5
	Foot Bath Combined With Acupoint Injection	5
	Topical Rubbing	5

**TABLE 3:** Top 5 oral Chinese medicines: introduction to medicinal plants.

Latin name	Bopomofo	Latin names of plants	Medicinal parts
Radix Astragali	Huangqi	Astragalus membranaceus (Fisch.) Bge. var. mongholicus (Bge.)Hsiao; Astragalus membranaceus (Fisch.)Bge	Root
Angelicae Sinensis Radix	Danggui	Angelicasinensis (Oliv.) Diels	Root
Paeoniae Radix Rubra	Chishao	Paeonia lactiflora Pall. ; Paeonia veitchii Lynch	Root
Pheretima	Dilong	Pheretima aspergillum (E.Perrier); Pheretima vulgaris Chen; Pheretima guillelmi (Michaelsen); Pheretima pectinifera Michaelsen	All
Chuanxiong Rhizoma	Chuanxiong	Ligusticum chuanxiong Hort	Rhizome
Carthami Flos	Honghua	Carthamus tinctorius L	Flower
Persicae Semen	Taoren	Prunus persica (L.) Batsch; Prunus davidiana (Carr.) Franch	Ripe Seed
Paeoniae Radix Alba	Baishao	Paeonia lactiflora Pall	Root
Cinnamomi Ramulus	Guizhi	Cinnamomum cassia Presl	Shoot
Zingiberis Rhizoma Recens	Shengjiang	Zingiber officinale (Willd.) Rosc	Rhizome
Jujubae Fructus	Dazao	Ziziphus jujuba Mill	Ripe Fruit
Asari Radix et Rhizoma	Xixin	Asarum heterotropoides Fr. Schmidt var. mandshuricum (Maxim.)Kitag.; Asarum sieboldii Miq.var.seou1ense Nakai、Asarum sieboldii Miq	Root and Rhizome
Glycyrrhizae Radix et Rhizoma	Gancao	Glycyrrhiza uralensis Fisch	Root and Rhizome
Tetrapanacis Medulla	Tongcao	Tetrapanax papyriferus (Hook.) K. Koch	stem pith
Angelicae Pubescentis Radix	Duhuo	Angelica pubescens Maxim. f. biserrata Shan et Yuan	Root
Taxilli Herba	Sangjisheng	Taxillus chinensis (DC.) Danser	Leafy Stem Branche
Eucommiae Cortex	Duzhong	Eucommia ulmoides Oliv	Bark
Achyranthis Bidentatae Radix	Niuxi	Achyranthes bidentata Bl	Root
Gentianae Macrophyllae Radix	Qinjiao	Gentiana macrophylla Pall.; Gentiana straminea Maxim.、Gentiana crassicaulis Duthie ex Burk.; Gentiana dahurica Fisch	Root
Poria	Fuling	Poria cocos (Schw.) Wolf	Mushroom Kernel
Saposhnikoviae Radix	Fangfeng	Saposhnikovia divaricata (Turcz.) Schischk	Root
Ginseng Radix et Rhizoma	Renshen	Panax ginseng C. A. Mey	Root and Rhizome
Rehmanniae Radix Praeparata	Shudihuang	Rehmannia glutinosa Libosch	Tuberous Root
Cinnamomi Cortex	Rougui	Cinnamomum cassia Presl	Bark

**TABLE 4:** Composition, therapeutic effects, and pharmacological actions of oral Chinese medicines.

Oral Chinese medicine	Botanical drugs included	Effect	Action	References
Huangqi Guizhi Wuwu Decoction	Radix Astragali; Cinnamomi Ramulus; Zingiberis Rhizoma Recens; Paeoniae Radix Alba; Jujubae Fructus	Improves coldness, numbness, and pain in the limbs	1.Improves neurological function to prevent diabetic peripheral neuropathy of STZ-induced diabetic rats by attenuating oxidative stress through Nrf2 and Bcl2 activation. 2.Upregulates the expression of β-catenin, cyclin D1, c-cmyc mRNA, downregulates the expression of DKK1 mRNA, and activates the classical Wnt signaling pathway to promote nerve fiber repair and regeneration 3.Prevents chronic OIPN by dynamically regulating intestinal flora homeostasis, thereby ameliorating intestinal barrier damage and reducing serum LPS and relevant inflammatory factor levels in the colon, serum, and DRG.	Zheng et al. (2019), Manman et al. (2022), Zhang et al. (2023)
Buyang Huanwu Decoction	Radix Astragali; Pheretima; Angelicae Sinensis Radix; Paeoniae Radix Rubra; Chuanxiong Rhizoma; Persicae Semen; Carthami Flos	Improves limb numbness	1.Regulates the production of inflammatory cytokines and promotes axonal regeneration after nerve transection of rats 2.promotes growth and differentiation of neural progenitor cells	Sun et al. (2007), Kim et al. (2020)
Danggui Sini Decoction	Paeoniae Radix Alba; Angelicae Sinensis Radix; Cinnamomi Ramulus; Asari Radix et Rhizoma; Tetrapanacis Medulla; Jujubae Fructus; Glycyrrhizae Radix et Rhizoma	Improves coldness, numbness and pain in the limbs	1.Downregulates the expression level of NF-κb protein and m RNA to protect neuronal cells	Cheng (2019)
Duhuo Jisheng Decoction	Angelicae Pubescentis Radix; Taxilli Herba; Eucommiae Cortex; Asari Radix et Rhizoma; Cinnamomi Cortex; Achyranthis Bidentatae Radix; Angelicae Sinensis Radix; Gentianae Macrophyllae Radix; Saposhnikoviae Radix; Rehmanniae Radix Praeparata; Chuanxiong Rhizoma; Ginseng Radix et Rhizoma; Poria; Paeoniae Radix Alba; Glycyrrhizae Radix et Rhizoma	Improves pain and coldness in limbs	Reduces serum MAD levels, elevates GSH-Px levels, improves oxidative stress, repairs the nerve conduction system	Jie et al. (2018)
Taohong Siwu Decoction	Persicae Semen; Carthami Flos; Angelicae Sinensis Radix; Rehmanniae Radix Praeparata; Paeoniae Radix Alba; Chuanxiong Rhizoma	Improves numbness and pain in limbs	1.Upregulates the expressions of autophagy markers (LC3-II/LC3-I and Beclin1) mitochondrial autophagy markers (Parkin and PINK1) after CIRI 2.Regulates the biosynthesis of phenylalanine, tyrosine, and tryptophan, as well as the metabolism of taurine, hypo, taurine, ascorbic acid, alginate, riboflavin, biotin, acid and proline, phenylalanine, and pyrimidine, improving the diversity and abundance and adjusting the structure of the gut microbiota, thereby alleviating the state of blood deficiency and blood stasis	Ji et al. (2022), He et al. (2023)

### 3.6 Duration of treatment

Of the 1,229 studies, 1,103 (89.75%) specified the duration of treatment, while 126 (10.25%) did not. Among the seven categories of interventions, Comprehensive TCM Nursing had the highest proportion of studies that did not specify the duration at 67.1%. Conversely, Herbal Injections (0%), Others (0%), and Proprietary Chinese Medicines (3.51%) had the lowest proportions of unspecified durations. Among the studies that specified the duration, Proprietary Chinese Medicines had the longest average treatment duration at 77.07 days, while Comprehensive TCM Nursing had the shortest at 31.67 days. See Table 5.

**TABLE 5:** Average duration of intervention and proportion of studies without specified duration.

Intervening measure	Oral Chinese medicine	External TCM therapies	Herbal injections	Proprietary Chinese medicines	Comprehensive TCM nursing	Multi-therapy combinations	Others	All interventions
Average Duration of Treatment (day)	53.39	32.23	32.05	77.07	31.67	40.26	75.20	44.49
Literature Quantity	451	379	20	57	73	244	5	1,229
The Number of Articles without Indicating the Duration of Treatment	24	34	0	2	49	17	0	126
The Proportion of Articles without Specifying the Duration of Treatment	5.32%	8.97%	0%	3.51%	67.12%	6.87%	0%	10.25%

### 3.7 Outcome measures

A comprehensive analysis of the outcome measures employed in the included RCT studies on TCM treatment for DPN was conducted. These outcome measures were categorized into 17 types, including neurophysiological evaluations, neuropathy scores, safety indicators, hemorheology, etc. Details for some of the outcome measure categories are provided in Table 6.

**TABLE 6:** Details of selected outcome measure categories.

Classification of evaluation indicators	The name of the evaluating indicator
Neurophysiological Evaluations	Motor Nerve Conduction Velocity (MCV), Sensory Nerve Conduction Velocity (SCV), Action Potential Amplitude, etc.
Neuropathy Score	Toronto Clinical Scoring System (TCSS), Toronto Clinical Neuropathy Score (TCNS), Michigan Diabetic Neuropathy Score (MDNS), Michigan Neuropathy Screening Instrument (MNSI), Clinical Symptom Integral Table (CSIT), Total Symptom Score-6 (NTSS-6), Neurological Disability Score (NDS), Neurological Symptom Score (NSS), Total Symptom Score (TSS), Leeds assessment of neuropathic symptoms and signs (S-LANSS),etc.
Safety Indicators	Incidence of Adverse Drug Reactions, Blood Routine, Liver Function, Renal Function, Electrocardiograph, etc.
Hemorheology	Plasma Viscosity, Whole Blood Relative Viscosity (WBRV), Whole Blood Low Shear Viscosity, Whole Blood High Shear Viscosity, RBC Aggregation Index, RBC Deformation Index (RDI), etc.
Indicators of Oxidative Stress	Serum Ferritin (SF), Malondialdehyde (MDA), Superoxide Dismutase (SOD), Glutathione Peroxidase (GSH-Px), Total Antioxidative Capacity (T-AOC), Advanced Glycation End Products (AGEs), Homocysteine (HCY), Cystatin C(Cys-c), etc.
Markers of Inflammation	Interleukin-6(IL-6), Interleukin-1β(IL-1β), Interleukin-18(IL-18), Free Fatty Acid (FFA), TNF-α, NF-κB, NLRP3 Inflammasome, High Mobility Group Box-1 Protein (HMGB1), Toll-like Receptor 2 (TLR2), Toll-like Receptor 4 (TLR4), Myeloid Differentiation Primary Response 88 (MyD88), Lp-PLA2, Prostaglandin F2α(PGF2),etc.
Function of Endothelial Cell	Nitric-oxide (NO), Endothelin (ET), Vascular Endothelial-Derived Growth Factor (VEGF), Nitric-oxide Synthase (NOS), etc.
Living Quality	36-Item Short Form (SF-36), Pittsburgh Sleep Quality Index (PSQI), Diabetes Specificity Quality of Life Scale (DSQL), Nottingham Health Profile (NHP), Sleep State Self-Rating Scale (SRSS), World Health Organization Quality of Life Assessment (WHOQOL-BREF), Activity of Daily Living (ADL), EORTC Core Quality of Life questionnaire (EORTC QLQ-C30), Barthel Index, EuroQol Five Dimensions Questionnaire (EQ-5D), etc.
Pain Score	Visual Analogue Scale (VAS), Numerical Rating Scale (NRS), Brief Pain Inventory for Diabetic Peripheral Neuropathy (BPI-DPN), the Neuropathy Pain Scale (NPS), etc.
Psychological Status	Self-rating Depression Scale (SDS), Self-Rating Anxiety Scale (SAS), Connor-Davidson Resilience Scale (CD-RISC), Diabetes Distress Scale (DDS), Hamilton Depression Scale (HAMD), etc.

Based on the assessment metrics utilized in the included RCT, studies on TCM, treatment for DPN, we categorized them into 17 types. The detailed contents for some of these categories are listed in Table 7.

**TABLE 7:** Evaluation metrics rankings by treatment approach.

Rank	Oral Chinese medicine metrics	External TCM therapies metrics
1	Neuroelectrophysiology	Efficacy Rate
2	Efficacy Rate	Neuroelectrophysiology
3	Symptom Sign	Symptom Sign
4	Traditional Chinese Symptoms	Neuropathy Score
5	Neuropathy Score	Traditional Chinese Symptoms
6	Blood Glucose	Safety Metrics
7	Safety Metrics	Blood Glucose
8	Hemorheology	Pain Rating
9	Oxidative Stress	Quality of Life
10	Inflammation Indicators	Other
11	Blood Lipids	Hemorheology
12	Endothelial Cell Function	Blood Lipids
13	Quality of Life	Inflammation Indicators
14	Other	Oxidative Stress
15	Pain Rating	Ankle-Brachial Index
16	Ankle-Brachial Index	Psychological Status
17	Psychological Status	Endothelial Cell Function

**FIGURE 4:**
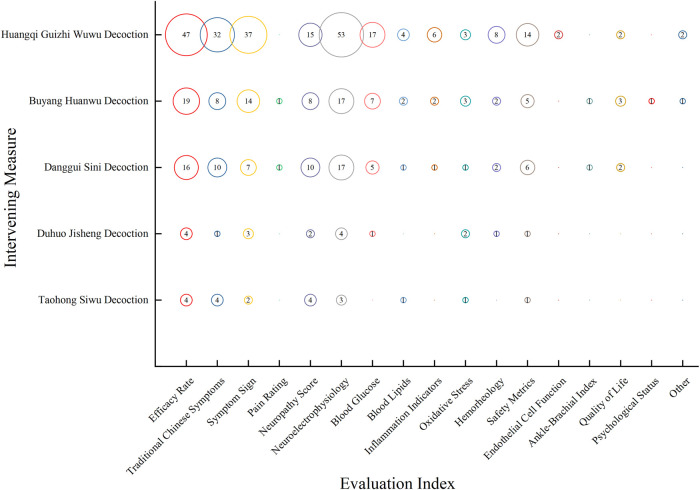
Distribution of evidence on evaluation metrics in clinical RCTs for the treatment of DPN with oral Chinese medicine over the last 6 years. The size of each circle represents the number of publications that employed a particular evaluation metric for this intervention.

Bubble diagrams indicate that clinical RCTs of common oral Chinese medicine treatments for diabetic peripheral neuropathy often focus on metrics such as Neuroelectrophysiology, Efficacy Rate, and Symptom Sign. In contrast, those metrics like Endothelial Cell Function, Quality of Life, and Ankle-Brachial Index receive less attention, as shown in Figure 4.

**FIGURE 5:**
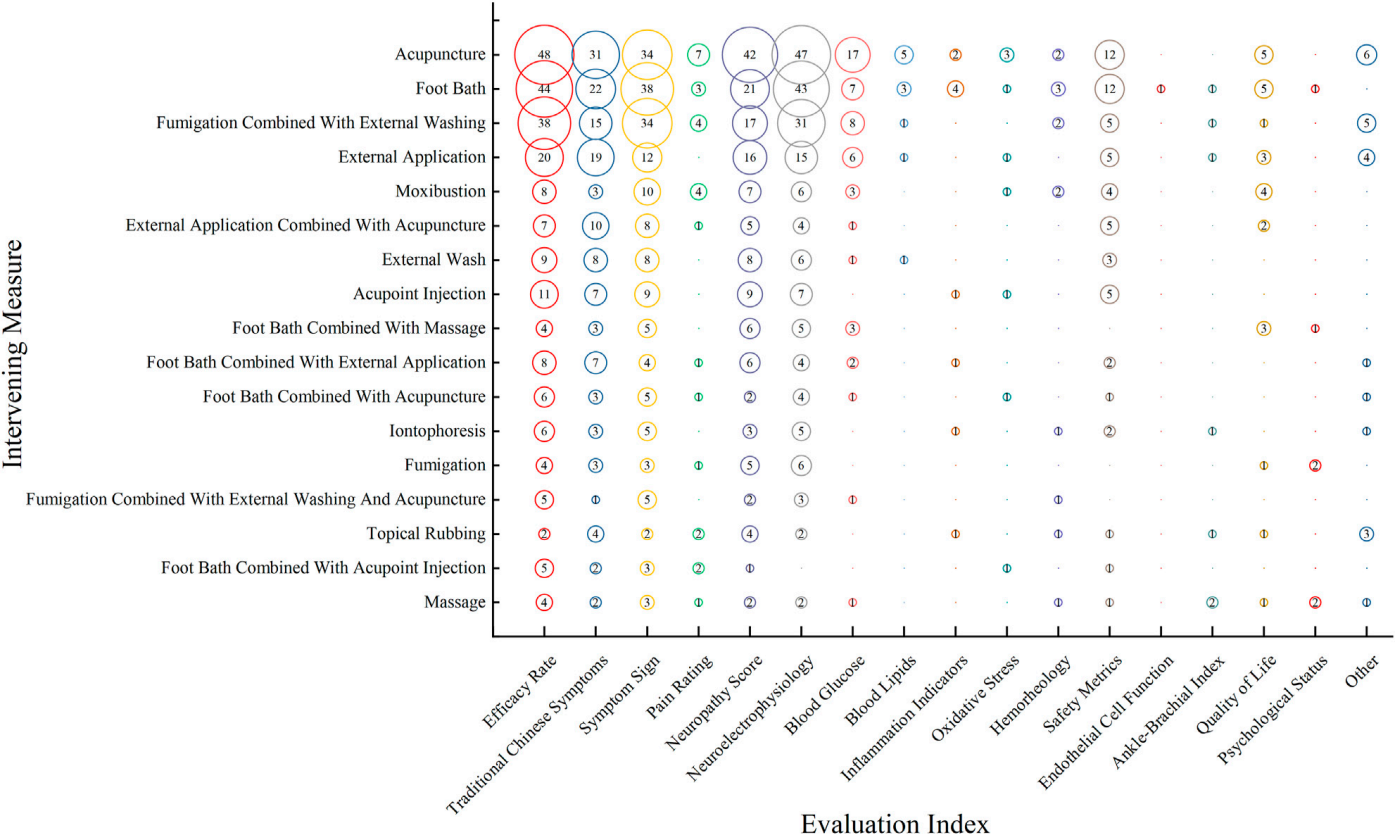
Distribution of evidence on evaluation metrics in Clinical RCTs for the treatment of DPN with TCM external treatment over the last 6 years. The size of each circle represents the number of publications that employed a particular evaluation metric for this intervention.

Clinical RCTs of prevalent TCM External Treatment pay more attention to Efficacy Rate, Neuroelectrophysiology, and Symptom Sign, while metrics such as Endothelial Cell Function and Psychological Status are often overlooked. Both treatment approaches prioritize Neuroelectrophysiology, Efficacy Rate, and Symptom Sign, with lesser emphasis on Ankle-Brachial Index and Psychological Status, as depicted in Figure 5.

## 4 Discussion

This study represents the first comprehensive examination of clinical RCTs regarding the TCM treatment of DPN over the past 6 years, using an evidence map approach. Through an in-depth analysis encompassing publication trends, study scale, positive drug usage, intervention categories, duration of treatment, and evaluation metrics, we shed light on the current evidence, research advancements, and challenges in TCM’s treatment of DPN. This review aims to guide future RCT research in this domain, assisting in refining both research focus and design.

### 4.1 TCM treatment for DPN: integrative trends and requirements

From the 1,229 RCT articles reviewed, the number of TCM studies addressing DPN has remained relatively stable over the past 6 years. Notably, up to 80% of these studies were of small scale, with sample sizes of fewer than 100 participants. Several studies displayed design flaws such as ambiguous sample sources, insufficient descriptions of randomization and blinding methods, a lack of baseline characteristics for trial participants, and an absence of drug washout periods, all of which could significantly influence the study outcomes.

However, concurrently, we observed a significant trend integration where nearly 70% of the interventions combined modern drugs like metformin, alpha-lipoic acid, and ipragliflozin with TCM. This combination potentially offers a dual therapeutic approach, fully harnessing the rapid and quantitative effects of modern drugs and the holistic and long-term benefits of TCM. For instance, while modern drugs might swiftly stabilize the blood sugar levels in diabetic patients, TCM can provide long-term health benefits by adjusting the overall balance in the body. Additionally, TCM often emphasizes moderating lifestyle habits, dietary practices, and emotional states, crucial aspects that might be overlooked in modern medicinal treatments.

Such integration underscores the advantages of combining traditional and modern medicine, offering patients a more comprehensive and integrative treatment modality. However, studies solely relying on traditional Chinese methods remain scarce. This may reflect an integration trend between TCM and contemporary medicine, suggesting an imperative for more exclusive TCM clinical trials to ascertain its efficacy. In the future, conducting large-scale, multi-center clinical randomized controlled trials would be valuable to confirm the effectiveness of TCM interventions. Such studies could compare TCM treatments with standard medications to assess non-inferiority, thus offering evidence to back the use of TCM as a standalone option in the prevention and management of diabetic peripheral neuropathy. (Hulley et al., 2017; Wang et al., 2022).

The inconsistency observed in the diagnostic criteria across RCT literature, with some studies providing clear guidelines while others merely confirming a diagnosis of diabetic peripheral neuropathy, underlines the necessity for uniform diagnostic standards in future research. To enhance the rigor and comparability of RCTs, it is crucial to use explicit diagnostic criteria. Researchers are encouraged to consult established guidelines, such as the American Diabetes Association’s position statement on Diabetic Neuropathy (Pop-Busui et al., 2017), the Standards of Medical Care in Diabetes-2020 (Association, 2020), and the Guideline for the prevention and treatment of type 2 diabetes mellitus in China (2020 edition) (Society, 2021), to ensure consistency and reliability in the field.

### 4.2 Diversity of outcome measures and the necessity of quantification in TCM syndrome diagnosis

A variety of efficacy evaluation indices were used, with DPN diagnostic-related indices, such as symptom and sign assessment, neurophysiological tests, and nerve lesion scoring (Fang et al., 2017), as well as TCM syndrome scores and overall efficacy rates being the primary ones. However, attention to the ankle-brachial index and psychological states was notably lacking. As a commonly used composite index, the efficacy rate is frequently applied based on the “Guidelines for Clinical Research of New Chinese Medicines” and “Standards for the Evaluation of TCM Syndromes” and combines various evaluation metrics, causing significant variability between studies and a lack of scientific rigor (Zhang et al., 2020). Future studies should opt for primary, singular symptoms or signs to determine relative objective evaluation criteria. It's also recommended that DPN researchers collaboratively explore a Core Outcome Set (COS) and develop common COS guidelines, aiming to simplify trial design, select efficacy evaluation indices, reduce outcome reporting bias, and minimize inter-study outcome reporting heterogeneity (Clarke, 2007; Yu et al., 2016).

Diagnosis and treatment in TCM are determined by a combination of disease diagnosis, symptoms, and syndrome identification. TCM syndrome is an essential aspect in addition to disease diagnosis and symptomatology. Issues with the TCM syndrome scoring system in RCT studies of TCM treatment for DPN include the lack of scientific quantification of symptom scoring and the subjective nature of efficacy judgments. It's suggested that future research could utilize a scoring system for syndrome efficacy metrics, based on the severity of DPN-related symptoms and signs and the contribution defined by TCM syndrome characteristics, thereby transforming TCM syndrome metrics into objective, quantifiable ones.

### 4.3 Comprehensive perspectives on DPN-related complications and TCM treatment

DPN and lower limb vascular lesions are pivotal contributors to diabetic foot ulcers. Their severity has been positively correlated (Song, 2018). The ankle-brachial index, a significant measure for evaluating lower limb arterial status, provides considerable prognostic insight for DPN patients (Hinchliffe et al., 2020; Sorber and Abularrage, 2021).

Interestingly, between 26% and 50% of DPN patients also report symptoms of anxiety or depression (Gore et al., 2005; Zhao and Qi, 2022). Persistent symptoms, such as abnormal limb sensations and functional impairments in DPN patients, increase their susceptibility to depressive states. Such depressive conditions not only intensify their pain and numbness but also diminish their motivation to control blood sugar, exacerbating their condition and creating a vicious cycle (DU Shun-Tang et al., 2022).

Several oral TCM medications have demonstrated efficacy in treating diabetes-associated depression by attenuating insulin resistance, mitigating oxidative stress, and modulating the nervous system (Lu et al., 2020). Furthermore, non-pharmacological TCM interventions like acupuncture and massage excel in alleviating pain, anxiety, and depressive symptoms, significantly enhancing patients’ quality of life (Yan, 2013; Wang et al., 2018). We recommend that future research on TCM for DPN broaden its scope beyond merely alleviating symptoms to encompass the overall quality of life and mental health of patients. This approach will contribute to a more holistic evidence base supporting the use of TCM in treating DPN.

Our findings indicate that non-pharmacological treatments like acupuncture and moxibustion, alongside oral Chinese medicine, are increasingly utilized for DPN management. We recommend that future research should focus on conducting more RCTs and meta-analyses specifically on these non-pharmacological Chinese medicine therapies, such as acupuncture for DPN. This approach will enable a more comprehensive evaluation of the effectiveness of non-pharmacological Chinese medicine treatments on DPN.

### 4.4 Study limitations and future directions

While this study provides substantial evidence for the clinical investigation of TCM in treating DPN, it is not without limitations:

The search scope was confined to clinical randomized controlled trials published in Chinese and English databases over the recent 6 years, resulting in a relatively narrow range of included study types. To gain a more comprehensive understanding of this field, future studies should consider broadening their search criteria to encompass various types of studies and explore other literature sources, such as clinical trial registration platforms.

In illustrating evidence distribution, interventions were categorized and distinguished based on type. This method’s shortcoming is that some intervention categories may encapsulate different drugs and operational methods. Furthermore, interventions within the same category might not be uniformly implemented, potentially influencing the research outcomes to varying degrees. No detailed quality assessment was carried out for the included studies, suggesting that the evidence distribution might be affected by biases to some extent.

Building on the previous discussion, we acknowledge the absence of a quantitative synthesis of data as a limitation. Future studies should incorporate quantitative analyses, such as meta-analyses, to provide more definitive conclusions regarding the effectiveness of TCM treatments for DPN. Such efforts will help clarify the magnitude of effects of various TCM treatments and identify promising approaches for further clinical investigation. By addressing these limitations and embracing a more comprehensive research methodology, future research can significantly advance our understanding and application of TCM in treating DPN.

## 5 Conclusion

This study systematically evaluated the clinical RCTs of TCM in addressing DPN, a significant complication of diabetes, using an evidence mapping approach. Despite the breadth of studies in this area, challenges such as inconsistent trial designs, predominant focus on standalone TCM treatments, varied intervention methods, and restricted evaluation metrics remain (Zhang et al., 2021).

To improve research quality regarding this pivotal diabetes complication, future studies should emphasize rigorous RCT design and apt selection of evaluation criteria. Moreover, establishing an online research database specifically tailored for TCM treatment of DPN—with a standardized literature extraction format—would be beneficial, promoting collaborative research and facilitating re-assessment of data. By integrating global research expertise, it's essential to advance the synergy of TCM and modern medicine, aiming to enhance therapeutic outcomes and better the quality of life for patients grappling with this complication.

Our research accentuates the profound role of TCM in tackling DPN as a notable diabetes complication, providing meaningful insights for informed medical decisions and optimized clinical practices. This investigation endeavors to bolster the scientific underpinning of clinical practices, aiming to elevate patient satisfaction and understanding of DPN within the larger context of diabetes-related complications.

In conclusion, the potential of TCM in addressing DPN as a significant diabetes complication is evident. We look forward to further research that explores its potential efficacy in this domain.

## Data Availability

The raw data supporting the conclusion of this article will be made available by the authors, without undue reservation.
